# Molecular Structure of Human-Liver Glycogen

**DOI:** 10.1371/journal.pone.0150540

**Published:** 2016-03-02

**Authors:** Bin Deng, Mitchell A. Sullivan, Cheng Chen, Jialun Li, Prudence O. Powell, Zhenxia Hu, Robert G. Gilbert

**Affiliations:** 1 School of Pharmacy, Tongji Medical College, Huazhong University of Science and Technology, Wuhan, Hubei, 430030, China; 2 Centre for Nutrition and Food Science, Queensland Alliance for Agriculture and Food Innovation, The University of Queensland, Brisbane, QLD, 4072, Australia; 3 Program in Genetics and Genome Biology, The Hospital for Sick Children, Toronto, ON, M5G 1X8, Canada; 4 Department of Endocrinology, Wuhan General Hospital of Guangzhou Military Command, Wuluo Road 627, Wuhan, Hubei, 430070, China; 5 Department of Plastic Surgery, Wuhan Union Hospital, Tongji Medical College, Huazhong University of Science and Technology, Wuhan, Hubei, 430030, China; University of Edinburgh, UNITED KINGDOM

## Abstract

Glycogen is a highly branched glucose polymer which is involved in maintaining blood-sugar homeostasis. Liver glycogen contains large composite α particles made up of linked β particles. Previous studies have shown that the binding which links β particles into α particles is impaired in diabetic mice. The present study reports the first molecular structural characterization of human-liver glycogen from non-diabetic patients, using transmission electron microscopy for morphology and size-exclusion chromatography for the molecular size distribution; the latter is also studied as a function of time during acid hydrolysis *in vitro*, which is sensitive to certain structural features, particularly glycosidic vs. proteinaceous linkages. The results are compared with those seen in mice and pigs. The molecular structural change during acid hydrolysis is similar in each case, and indicates that the linkage of β into α particles is not glycosidic. This result, and the similar morphology in each case, together imply that human liver glycogen has similar molecular structure to those of mice and pigs. This knowledge will be useful for future diabetes drug targets.

## Introduction

Liver glycogen is important in maintaining blood-glucose homeostasis. It is a highly branched polymer of glucose which also contains small but significant amounts of protein [1–3]. While glycogen is also present in heart, skeletal muscle, brain and adipose tissues, glycogen in the liver is responsible for acting as our blood-glucose buffer [4]. Liver glycogen has three levels of structure: 1) glucose units are attached to form linear chains via α-(1→4) linkages; 2) these chains are joined together via α-(1→6)-linked branch points to form highly branched glycogen “β particles” (~20 nm in diameter); and 3) these β particles are able to be joined together (perhaps by a bond involving a protein) to form much larger “α particles” (up to 200 nm in diameter) [5], which have a composite cauliflower-like appearance under transmission electron microsopy (TEM).

Due to the obvious ethical and practical limitations of performing research on human liver samples, the use of animal models has often been necessary for diabetes research. The translation of this research into human-liver tissues will enhance the physiological relevance of discoveries made with animal models.

A recent study analyzing the molecular size distributions of glycogen from diabetic (*db/db*) and non-diabetic (*+/db*) mouse livers found that the glycogen extracted from diabetic livers is significantly more fragile than that of the non-diabetic controls [6, 7]. Specifically, the composite α particles in diabetic liver, while similar in amount and size distribution to those in healthy liver, readily fragment into β particles in dimethyl sulfoxide (DMSO), a solvent which is chemically inactive to glycosidic bonds but which breaks hydrogen bonds. Given the evidence that larger glycogen α particles enzymatically degrade to glucose more slowly than the smaller β particles, together with the characteristically poor control of blood glucose in type 2 diabetics, it is reasonable to postulate that the fragile nature of diabetic glycogen may exacerbate the pathology of the disease [8–10]. Human studies are therefore highly desirable. Ethical considerations however preclude obtaining human-liver glycogen samples by biopsy from diabetic individuals who have had no treatment for the disease.

The mechanism whereby β particles join together to form α particles is still unknown. Our previous results indicate that this linkage involves a protein, as yet unidentified but possibly glycogenic or lectin. However, human and mice might have the same protein but different isoforms. For example, glycogenin has two isoforms: glycogenin-1 and glycogenin-2. Mice only have glycogenin-2 but humans have both. If a glycogenin is indeed the linkage, it is of interest to see if human-liver glycogen has similar molecular structure to that in mice. This will provide some new knowledge about the formation of α particles.

Here we report the first molecular structural studies of human-liver glycogen, analyzing the molecular structure in both hepatitic and non-hepatitic liver. The objective is to see if there are indications of structural similarity in the α and β particles of non-diabetic human and mouse glycogen. If strong similarities were to be found, this would support the hypothesis that inferences from studies on diabetic mice may well also apply to humans, while if this were not to be the case, it would refute the hypothesis.

The following methods are used here for structural characterization. (1) Size-exclusion chromatography (SEC, also denoted GPC or HPLC-SEC), which separates fully dissolved and dispersed molecules by size (specifically, by the hydrodynamic volume or hydrodynamic radius, *R*h); with refractive index detection, this yields the weight distribution of molecules as a function of *R*h. (2) TEM for morphology. (3) Acid hydrolysis with measurement of molecular size distribution as a function of hydrolysis time, which yields information on the binding joining the β particles into α particles [11]. These data are sensitive to the nature of bonding, particularly glycosidic vs. proteinaceous linkages: the former degrade very slowly under the conditions used here, the latter degrade very gradually. Acid hydrolysis as a structural investigative tool is quite different from the large body of work involving glycogenolysis inhibitors. These simply suppress glycogen degradation and increase glycogen content, which may or may not affect the structure of glycogen. Their use would not be of assistance in our goal of investigate the binding of α particles in human-liver glycogen

## Materials and Methods

### Animal tissue

This study was carried out in strict accordance with the ‘Animal Research: Reporting in Vivo Experiments’ (ARRIVE) guidelines. The protocol was approved by the Huazhong University of Science and Technology Tongji Medical College Animal Care and Ethics Committee. All surgery was performed under sodium pentobarbital anesthesia, and all efforts were made to minimize suffering. Female mice (C57BL/6J) were purchased from the Model Animal Research Center of Nanjing University. These mice were housed in standard cages (3–6 mice per cage), with the temperature controlled at 22 ± 1°C. A 12 h dark/light cycle was used, with lights on at 7 am. Mice had *ad libitum* access to standard chow (6% kcal from fat, 14.3 MJ kg^-1^, Hubei Provincial Center for Disease Control and Prevention) and water. At 12 weeks of age (equivalent to young-middle age in humans; previous work [6, 7] showed no significant change with age in liver-glycogen size distribution in mice), mice were divided into two groups. One group of mice had *ad libitum* access to food, another group of mice were fasted 12 h before being sacrificed. Then mice were anaesthetized at approximately 9 am with sodium pentobarbitone (150 mg kg^–1^, i.p.), with their livers being rapidly excised and snap frozen in liquid nitrogen. Samples were stored at –80°C.

### Human tissue

Human liver-tissue was obtained from the Wuhan General Hospital of Guangzhou Military. This conformed to the ethical guidelines of the 1975 Declaration of Helsinki as reflected in *a priori* approval by the Human Research Committee of Huazhong University of Science and Technology and Wuhan General Hospital of Guangzhou Miltary Command. Patients gave written consent as part of their consent to undergo surgery. All were fasted for at least 8 h before surgery. The tissue was taken for further pathological examination from patients during surgery and was snap-frozen in liquid nitrogen. Informed consent was received from each patient. Information on each of the 10 human patients is given in Table 1. None had diabetes or insulin resistance.

**Table 1:** Information of patients.

Sample	Gender	Age	Disease	Part of liver extracted
**1**	M	49	Liver cancer and hepatitis B	Hepatitis B
**2**	F	52	Hepatic hemangioma	Tumour
**3**	F	50	Intrahepatic stones	Healthy
**4**	M	49	Liver cancer and hepatitis B	Tumour
**5**	F	69	Intrahepatic stones	Healthy
**6**	F	52	Intrahepatic stones	Healthy
**8**	F	48	Intrahepatic stones	Healthy
**11**	M	56	Liver abscess	Liver abscess
**14**	F	54	Hepatic adenoma	Healthy
**15**	F	60	Intrahepatic stones	Healthy

### Glycogen extraction

Liver glycogen from both mice and humans was extracted as in a previous study [8]. Approximately 1 g of liver was homogenized in 25 mL of glycogen isolation buffer (50 mM Tris, pH 8, 150 mM NaCl, 2 mM EDTA, 50 mM NaF and 5 mM sodium pyrophosphate). 200 μL of this homogenate was removed for glycogen content determination. Samples were centrifuged at 6000 *g* for 10 min at 4°C. The supernatants were then centrifuged at 260 000 *g* for 2 h at 4°C. The pellet was then resuspended in glycogen isolation buffer and layered over a 20 mL stepwise sucrose gradient (37.5% and 75% in deionized water). These samples were then centrifuged at 370 000 *g* for 2.5 h at 4°C. The pellet of glycogen at the bottom of the tube was resuspended in 0.5 mL of deionized water. Samples were mixed with 4 parts absolute ethanol to precipitate the glycogen The samples were then centrifuged at 4000 *g* for 10 min and the pellets were re-dissolved in 1 mL of deionized water and lyophilised (freeze-dryer; VirTis, BTP-9EL).

### Glycogen content determination

The glycogen content of each liver specimen was determined as previously employed [8, 12]. This method uses amyloglucosidase to enzymatically degrade glycogen into glucose, followed by glucose content measurement using a glucose oxidase/peroxidase (GOPOD, Megazyme) assay kit. Briefly, 5 μL of amyloglucosidase (3260 U mL^–1^ on soluble starch, Megazyme), 20 μL of homogenate resulting from the extraction, and 100 μL of sodium acetate buffer (pH 4.5) was made up to 500 μL with deionized water and incubated for 30 min on a thermomixer at 50°C. A control containing everything except amyloglucosidase was also analysed. An aliquot of 300 μL from each sample was added to 1 mL of GOPOD reagent and incubated at 50°C for a further 30 min on a thermomixer. The absorbance of each sample (510 nm) was analysed using a UV-6100s MAPADA. The glycogen content was calculated by constructing a calibration curve with D-glucose reacted with the GOPOD reagent. All samples and controls were run in duplicate with the average values being used.

### Size exclusion chromatography of glycogen

Size exclusion chromatography (SEC) analysis was performed with a technique used previously [13]. Glycogen (2 mg mL^–1^) was dissolved in a thermomixer for 8 h at 80°C in 50 mM ammonium nitrate/0.02% sodium azide. Samples were injected into an Agilent 1260 Infinity SEC system (Agilent, Santa Clara, CA, USA) using a SUPREMA pre-column, 1000 and 10000 columns (Polymer Standard Service, Mainz, Germany). The columns were kept at 80°C using a column oven and the flow rate was set to 0.3 mL min^–1^. A refractive index detector (Optilab UT-rEX, Wyatt, Santa Barbara, CA, USA) was used to determine the SEC weight distributions. Pullulan standards (PSS), with a molar mass range of 342–2.35 × 10^6^ Da, were dissolved in 50 mM ammonium nitrate/0.02% sodium azide and run through the SEC system, allowing the construction of a universal calibration curve.

### Transmission electron microscopy (TEM) of glycogen

Glycogen was resuspended in 50 mM Tris-buffered saline pH 7.0 at a concentration of 1 mg mL^–1^. The suspension was then diluted 10-fold and applied onto a glow-discharged copper grid (400 mesh). After 2 min, excess sample was drawn off with filter paper, and the grids stained with two or three drops of 1% uranyl acetate. The preparations were examined using a Hitachi H-7000 transmission electron microscope operating at 75 kV using AnalySiS image management software.

### Acid hydrolysis of glycogen

An acid hydrolysis method slightly modified from that used previously was applied here [14]. Glycogen (~2 mg mL^–1^) was dissolved in 0.1 M sodium acetate buffer (pH 3.5) and heated at 80°C in a thermomixer for 10 min, 30 min, 2 h and 12 h. Samples were removed from the thermomixer after acid hydrolysis and precipitated with four volumes of absolute ethanol. Samples were then centrifuged at 4000 *g* for 10 min and the pellets were re-dissolved in 1 mL of deionized water and lyophilised (freeze-dryer; VirTis, BTP-9EL). The dried samples were dissolved in 50 mM ammonium nitrate/0.02% sodium azide (the aqueous eluent of SEC) and run on the SEC.

## Results and Discussion

**Fig 1:**
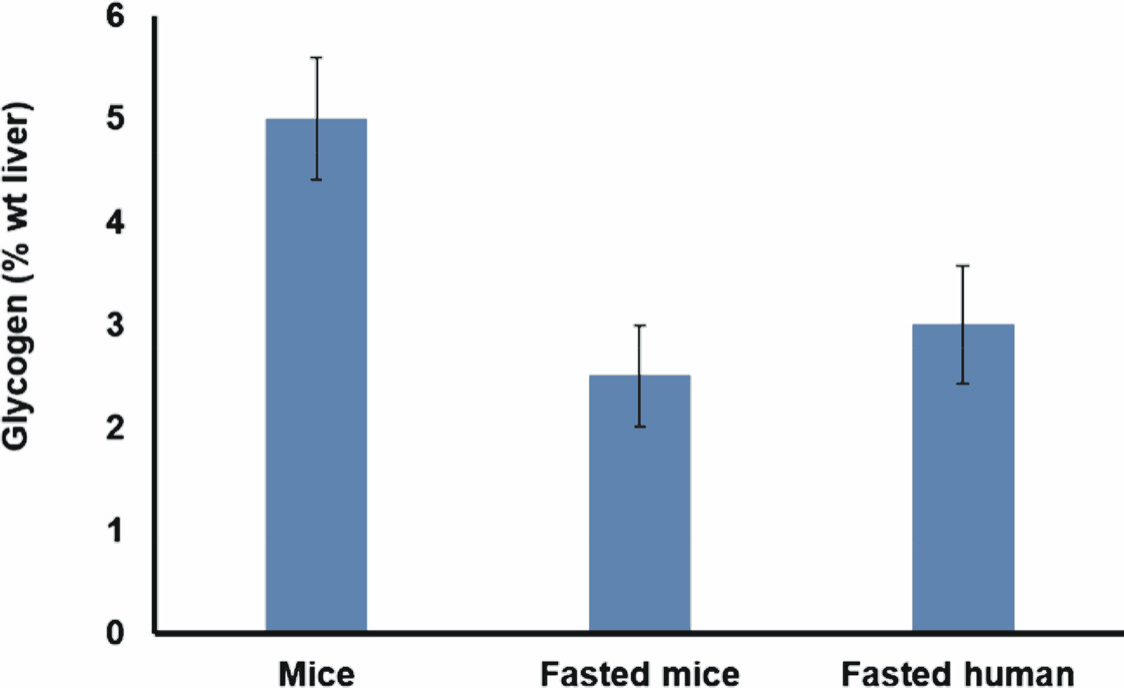
The glycogen content of mouse (sacrificed ~2 h after their last meal, and fasted for 12 h) and fasting human liver. The values shown are the mean ± standard error of mean (6 mice and 10 humans).

This publication focuses on finding any structural similarities between human- and mouse-liver glycogen. The glycogen contents of human and mouse liver are given in Fig 1. While the human patients had fasted for at least 8 h before surgery, their liver tissue still contained a significant amount (~3%) of glycogen. The glycogen content of mice sacrificed ~2 h after their last meal should be close to maximum levels (the end of the dark period [8]), was unsurprisingly relatively high at ~5%.

**Fig 2:**
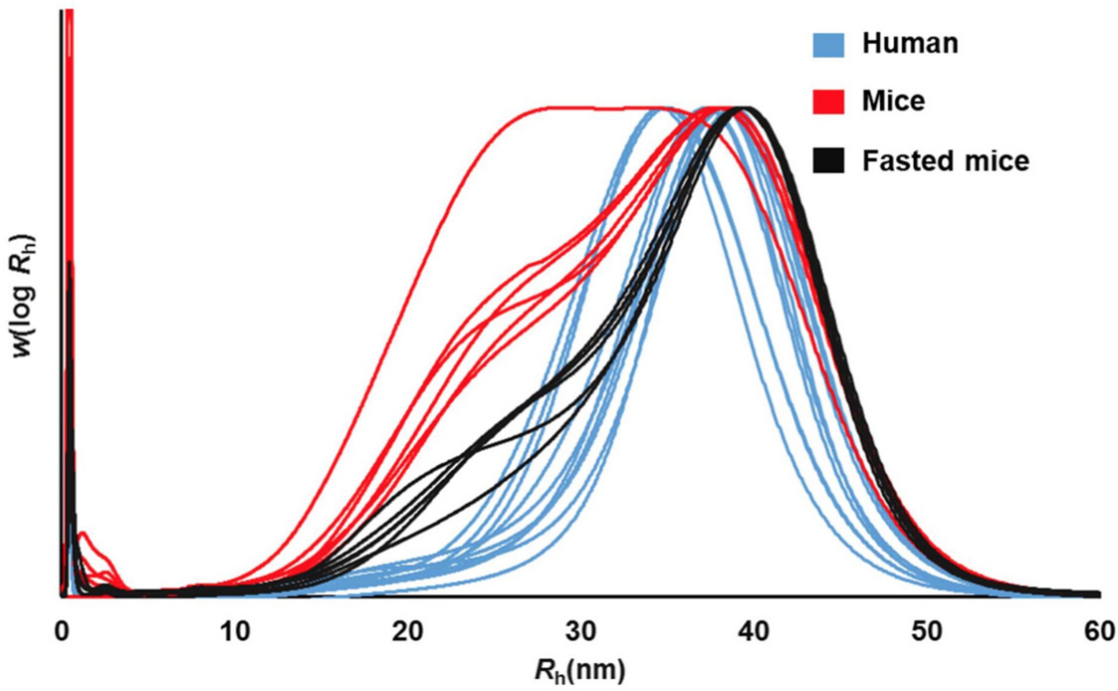
SEC weight distributions, *w*(log *R*_h_) (arbitrary units), for human-liver glycogen (blue; during surgery following ~12 h fasting) and mouse-liver glycogen (red, at end of dark period, this being just after last meal; black, fasted over 12 h before being sacrificed). Curves have been normalized to equal maximum heights.

In the last step of glycogen extraction and purification, four times absolute ethanol was added to precipitate glycogen. This step may denature the protein in glycogen but it would not change its size distribution. The SEC weight distributions of all human- and mouse-liver glycogen samples are given in Fig 2.The α-particle peaks of the human-liver glycogen are very similar to those in mice, with a maximum at *R*_h_ ~40 nm. However, while mouse-liver glycogen can have both β- and α-particle peaks [7, 13, 15], the human samples distributions in the present study are monomodal, containing only α particles. Whether this absence of β particles is characteristic of human-liver glycogen or is peculiar to the conditions required for surgery, especially 12 h fasting, is not clear. To test which of these two possibilities is applicable would require finding human liver that has been obtained without the normal requirements for pre-surgery fasting, which would involve atypical conditions, e.g. urgent surgery following major trauma. However, the lack of β particles after moderate fasting is consistent with a study performed involving mice sacrified at various stages of their eating cycle [8]. It was observed that mice towards the end of the fasting phase of their cycle contained predominantly α particles, a finding consistent with evidence that the smaller glycogen β particles degrade more rapidly (per glucose unit) than do α particles [9, 10]. Confirming this, the SEC distributions (see Fig 2) of mice which had undergone extensive fasting consists almost entirely of α particles, the same as seen in fasting humans. This similarity is quantified by determining the maximum *R*h and full-width at half-height (FWHH) of each individual distribution, and then finding the averages, which are as follows. Fasting human: *R*h(max) = 37.1±1.6 nm, FWHH = 11.2±0.8; fasting mice: *R*h(max) = 38.1±0.3 nm, FWHH = 13.6±1.0.

**Fig 3:**
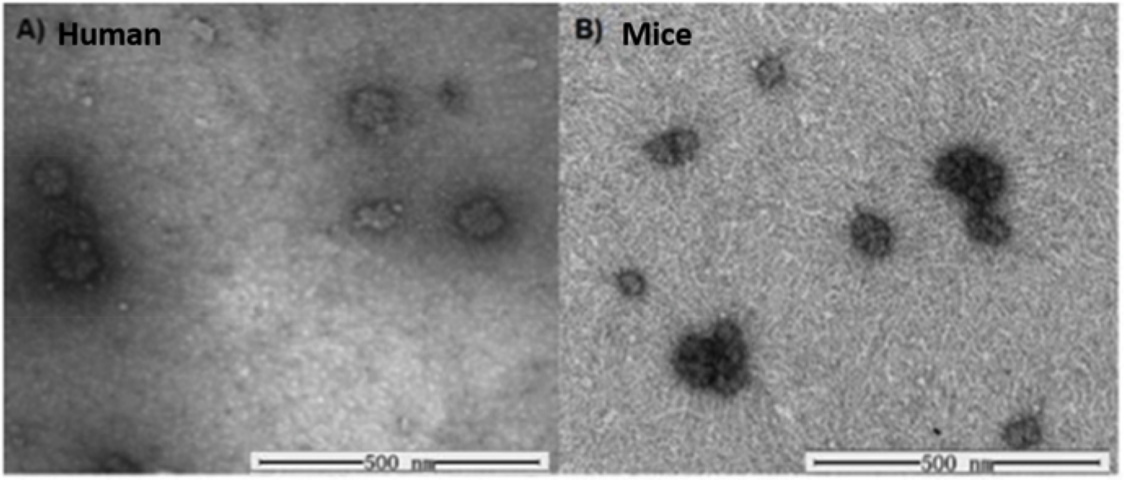
TEM images of human (A) and mouse (B) liver-glycogen.

Transmission electron spectroscopy (TEM) images of human- and mouse-liver glycogen are given in Fig 3. It can be seen in the TEM image that human and mice liver glycogen α particles have similar diameter, ~100 nm. The similar size in TEM images, while qualitative, is also quantitatively reflected in similar weight distributions.

### Acid hydrolysis of human α particles

One benefit from the human-liver glycogen samples containing only α particles is that for the first time we can test the effect of acid hydrolysis on a sample containing only α particles. While we have been able to infer important information on the degradation of α particles under acidic conditions with samples containing both α and β particles [11, 14], using a sample with only α particles allows us to directly observe how they degrade without any interference from β particles. We can also see whether human α particles under acid hydrolysis react similarly to the pig-liver glycogen that was used previously [11, 14].

**Fig 4:**
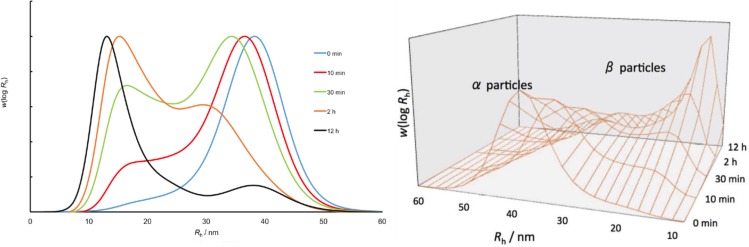
Time evolution of human-liver glycogen exposed to acidic conditions (pH ~3.5) for 0, 10, 30 min, 2 and 12 h. Left-hand panel: normalized to maximum of 1, as 1D plots; right-hand panel, normalized to total area, as 2D plot.

Fig 4 shows the SEC weight distributions as functions of size, with the same data shown in two different ways: normalized to a maximum of 1, and normalized to total area under the curve of *w*(log *R*h). It is seen that the degradation of human liver-glycogen is consistent with past studies that observed the larger α particles degrading into separate β particles [11, 14]. As stated, the starting material contains only α particles, which was different from the mixtures of α and β particles used in the previous studies. It is seen that a new peak appears, which is at about the size seen in a previous study [13] for β particles. The α-particle peak diminishes correspondingly; the position of its maximum appears to decrease slightly, then appears to increase in time, but this apparent decrease might be due to overlap with the β particle distribution. The rapid degradation of the α-particle peak is consistent with proteinaceous rather than glycosidic bonding between the component β particles.

This result is consistent with that seen for size distributions in pig-liver glycogen [11] except that the latter samples also contained significant amounts of β particles in the starting material. If the bond joining β particles together to form α particles was not more acid-labile than the glycosidic linkages holding glycogen together, then the single α-particle peak would decrease over time but remain monomodal, as is seen when phytoglycogen is acid-hydrolyzed [11]. This similarity in the time evolution of size distributions of human and pig glycogen under acid hydrolysis suggests that the binding holding β particles together in human-liver glycogen are similar to those in other mammals.

There is an interesting comparison with aspects of related previous work. The composite α particles in animal liver glycogen are also seen in phytoglycogen from mutant plants; as discussed elsewhere [11, 16], the presence of β particles of approximately the same size in each case is consistent with the growth of these β particles being limited by steric hindrance as the randomly-branched glycogen particle grows larger [17]. Besford et al. [18] examined both β and α particles using dynamic light scattering (DLS) and SAXS. While there are some questions about the DLS because of the problems with the high-angle scattering used there (because of the presence of multiple modes of motion as well as brownian motion of the whole particle [19, 20]), the hydrolysis data (with both acid and γ amylase, which both cleave randomly) are consistent with those from Powell *et al*.

The conclusion that the binding between β particles to form composite α particles in non-diabetic livers is similar in all the mammals considered here (human, mouse and pig) suggests that it is not unreasonable to make similar inferences about diabetic liver glycogen. Previous mouse studies [6–8] indicate that this bonding is impaired (fragile) in diabetic mice liver, and the degradation rate of big α particles is much smaller than that of β particles. This might contribute the hyperglycaemia in diabetic mice. If this inference is correct and the impaired binding also exists in diabetic human liver, this will shed some new insight about the pathogenesis of human diabetes.

## Conclusions

The inferences from the results, showing that animal studies are also relevant to human diabetes, are summarized as follows.

- Liver glycogen from fasting humans shows the same composite α particle morphology (made up of joined smaller β particles) as seen in that of other animals.
- The SEC weight distributions of liver glycogen from mice, pigs and fasting humans all show a maximum for the α-particle component at about the same size, and the distribution for fasting mice and fasting humans show a single α-particle peak.
- The time evolution of the size distribution during acid hydrolysis of human-liver glycogen is consistent with that observed elsewhere for other mammals.

This is the first study of the molecular structure in human-liver glycogen. Under acid hydrolysis, the evolution of the size distributions in humans and mice are similar, suggesting that the linkage of β into α particles is not glycosidic. This result, and the similar morphology in each case, together imply that human liver glycogen has similar molecular structure to that of mice. This is consistent with, but does not prove, the hypothesis that liver glycogen in diabetic humans will have the same structural impairment as in mice, an inference which cannot be investigated directly because of ethical considerations. Had a different result been found, this would have disproved this hypothesis. This knowledge will be useful for future diabetes drug targets.

## Supporting Information

S1 Data Data files for size distributions. (ZIP)
